# Severe Thrombocytopenia Associated With Black Seed Oil and Evening Primrose Oil

**DOI:** 10.7759/cureus.8390

**Published:** 2020-06-01

**Authors:** Xuejun Wang, Alicia Jiang, Vivek Batra

**Affiliations:** 1 Internal Medicine, Thomas Jefferson University Hospital, Philadelphia, USA; 2 Medical Oncology, Thomas Jefferson University Hospital, Philadelphia, USA

**Keywords:** nigella sativa, supplements, consumptive thrombocytopenia, drug-induced thrombocytopenia, primrose oil, black seed oil, postoperative thrombocytopenia

## Abstract

We report the case of a 69-year-old female with stage IIIB endometrial adenocarcinoma who developed an acute thrombocytopenia with greater than 90% decrease from her baseline value in platelets one day after a laparoscopic hysterectomy. Subsequently, the patient was found to have bilateral subsegmental pulmonary emboli and a right atrial thrombus. The thrombocytopenia reached a nadir of 31,000/mL^3^ from a baseline of 410,000/mL^3^ and resolved without intervention. Prior to the surgery, the patient was taking black seed oil, which is commonly used for its anti-carcinogenic effects, and evening primrose oil daily for approximately one month. A literature review revealed that black seed oil contains thymoquinone, which is a compound related to quinine. Evening primrose oil is also known to reduce platelet aggregation and has anti-thrombotic properties. We believe the patient’s thrombocytopenia was caused by a consumptive coagulopathy due to the formation of multiple thrombi and exacerbated by the use of herbal supplements, namely black seed and evening primrose oil.

## Introduction

Thrombocytopenia is defined as a platelet count < 150,000/mL^3^ (normal range: 150,000-450,000/mL^3^), and is classified as mild (platelet count 100,000-150,000/mL^3^), moderate (50,000-99,000/mL^3^), or severe (< 50,000/mL^3^). The major complication of thrombocytopenia is bleeding. Spontaneous bleeding typically occurs when platelet counts are < 10,000/mL^3^. However, the correlation between bleeding and platelet count is highly variable. Etiologies of thrombocytopenia can be broadly categorized into decreased platelet production, increased platelet consumption, and sequestration of platelets [1]. 

The treatment of thrombocytopenia involves addressing the underlying cause. In deep venous thrombosis (DVT) and pulmonary embolism (PE), the mechanism of thrombocytopenia is related to increased platelet consumption due to the formation of thrombi. In drug-induced immune thrombocytopenia (DITP), medications or supplements lead to the production of antiplatelet antibodies and result in increased platelet consumption, and treatment typically involves discontinuing the offending drug. Once the drug is stopped, platelet counts typically recover within one to two weeks without further intervention.

Although there is no evidence for treating DITP with immunosuppressive therapy such as intravenous immunoglobulin (IVIG) and steroids, in clinical practice, IVIG and steroids are frequently given when there is concern for bleeding because immune thrombocytopenia (ITP) is difficult to differentiate from DITP. Furthermore, platelet transfusions are given to prevent bleeding if platelets are <10,000/mL^3^ and <50,000/mL^3^ for surgical procedures. In this case report, we present a patient with significant acute postoperative thrombocytopenia after using black seed oil and primrose oil daily for one month. This case report highlights thrombocytopenia as a potential adverse side effect of black seed and primrose oil supplements [2,3]. Written informed consent was obtained from the patient for publication of this case report.

## Case presentation

The patient is a 69-year-old African American female who was transferred to our facility for the management of anticoagulation in the setting of new bilateral PEs and thrombocytopenia. Prior to the transfer, the patient underwent a total abdominal hysterectomy with bilateral salpingo-oophorectomy for suspected endometrial cancer. She was subsequently diagnosed with stage IIIB serous endometrial adenocarcinoma based on surgical pathology. Her postoperative course was complicated by a profound thrombocytopenia and bilateral subsegmental PE without right ventricular strain. Her prior medical history includes a previous right lower extremity DVT with a right lower lobe PE. They occurred two months before her endometrial carcinoma diagnosis, likely in the context of a hypercoagulable state. She had an inferior vena cava (IVC) filter placed and was eventually transitioned to apixaban for anticoagulation. Prior to the surgery, her home medications included apixaban 5 mg twice a day, amlodipine 10 mg daily, and acetaminophen 325 mg as needed for pain. She also took herbal supplements daily, including black-seed oil and primrose oil, for approximately one month prior to the surgery.

The patient had a baseline platelet count of 410,000/mL^3^, which was measured two weeks prior to the surgery, and a baseline hemoglobin of 12 g/dl. The patient had no medication or clinical changes between her last lab draw and her surgical procedure. Given that her most recent labs were stable and the patient had no changes during the time frame, there was minimal concern for lab abnormalities and labs were not drawn just prior to surgery. After the surgical procedure, her platelet count decreased to 37,000/mL^3^ on postoperative day 1. The estimated blood loss during the surgery was 600 mL, for which she was transfused 2 units of packed red blood cells and 1 unit of pooled platelets. However, the patient continued to have worsening thrombocytopenia after the surgery, with a nadir of 31,000/mL^3^ on postoperative day 1.

Anticoagulation was held until her platelets increased to 60,000/mL^3^ on postoperative day 5. Initially, she was placed on a heparin drip and then transitioned to therapeutic enoxaparin for long-term anticoagulation. Her platelets returned to her previous baseline of 400,000/mL^3^ in approximately two weeks without any additional intervention.

Her thrombocytopenia was extensively worked up and multiple etiologies of secondary thrombocytopenia were considered, including heparin-induced thrombocytopenia, hemolysis, and viral-associated and thyroid-related etiologies. The results of the work-up are outlined in Table 1. Her lab results were significant for postoperative hemoglobin of 10.5 g/dL and white blood cell count of 11.4 per microliter. 

**Table 1:** Work-Up for Secondary Thrombocytopenia

Study	Result
Heparin-induced thrombocytopenia work-up	
Serotonin-release assay	Non-reactive
Platelet factor 4	Non-reactive
Peripheral smear	No schistocytes
Haptoglobin	194 mg/dl
Lactate dehydrogenase (LDH)	291 units/L
Disseminated intravascular coagulation (DIC) panel	Negative
Fibrinogen	329 mg/dl
D-dimer	33,000 ng/ml
Antiphospholipid syndrome (APLS) work-up	
Anticardiolipin antibodies	Negative
B2 glycoprotein antibodies	Negative
Dilute Russell’s viper venom time (dRVVT)	Negative
HIV screen	Negative
Hepatitis B screen	Negative
Hepatitis C screen	Negative
Thyroid-stimulating hormone (TSH)	1.64 mIU/L

## Discussion

In this case, the patient had an acute decrease in platelets from a baseline of 410,000 platelets/mL^3^ prior to surgery to a nadir of 31,000 platelets/mL^3^ on postoperative day 1. The patient had started taking black seed and primrose oil supplements about one month prior to the surgery. Her recorded baseline of 410,000 platelets/mL^3^ occurred two weeks after she started taking those supplements. Given the patient’s work-up for secondary thrombocytopenia was negative, multiple etiologies could have contributed to this patient’s thrombocytopenia in the postoperative setting. Postoperative thrombocytopenia and consumptive thrombocytopenia from the formation of multiple venous thromboembolisms likely caused the patient’s acute thrombocytopenia. However, animal studies have also shown that ingestion of black seed oil or evening primrose oil decreased platelet counts in laboratory settings [2,3]. It is possible that the herbal supplements also partially contributed to the patient’s thrombocytopenia following her surgery.

Surgery alone can cause thrombocytopenia via endothelial cell dysfunction and tissue factor and von Willebrand factor release leading to platelet activation and consumption. Early-onset postoperative thrombocytopenia is usually mild and resolves spontaneously within three to four days without bleeding [4]. One study that evaluated platelet counts in patients undergoing orthopedic surgery found that 28% of patients became thrombocytopenic after surgery, with only one patient having a platelet level <100,000/mL^3^ postoperatively [5].

Acute thrombocytopenia can also occur from consumption related to an acute thrombus within 12 hours. An acute decrease in platelet count occurs following a DVT/PE because fresh thrombi exude thromboplastic substances and cause platelets to adhere to the thrombi’s surface. The thrombocytopenia typically resolves spontaneously within three days [4]. In one prospective study of 189 patients with acute PE, the mean platelet decrease was 33,000/mL^3^, from 290,000-256,000/mL^3^ [6]. Similarly, the Urokinase Pulmonary Embolism Trial found the mean platelet count in acute PE patients was 293,000/mL^3^ [7]. Another study enrolling patients with acute PE found admission platelet count to be 221,000/mL^3^ [8]. In contrast, one study found that patients who developed postop DVT, but not PE, had higher platelet counts than their preoperative levels [9].

Nigella sativa, or black-seed oil, is a popular herbal supplement that is frequently marketed as a “miracle supplement”. It has been studied for its beneficial effects, including anti-carcinogenic, anti-oxidant, anti-inflammatory and analgesic, anti-diabetic, and antimicrobial effects [10]. Side effects of Nigella sativa are understudied, though the few studies available have shown low toxicity [11]. The main component of Nigella sativa is thymoquinone, a quinine constituent [10,11]. Quinine is a well-known cause of DITP, and there are many case reports of thrombocytopenia associated with quinine-containing beverages and over-the-counter medications, as well as a systematic review that revealed those who experienced DITP secondary to quinine had a median nadir platelet count of 3,000/mL^3^ [12-14]. Animal studies on black-seed oil’s effects on platelets have shown that oral administration of the fixed oil for 12 weeks decreased platelet counts by approximately 15%-35%, compared to control values [2]. Another study proposed that thymoquinone induces apoptosis of platelets cells via a GPCR family receptor [15]. 

A wide variety of agents, including medications and many herbal substances, can cause the secondary form of ITP referred to as DITP. In addition to quinine, an analysis of case reports found 25 herbal substances and alternative medicines associated with thrombocytopenia. Many of the case reports reported platelet values as low as 0-16,000/mL^3^, although many of the patients did not experience thrombocytopenia alone [13]. The clinical course of DITP involves platelet counts decreasing in a week or longer after the drug is initiated depending on the frequency and quantity used [16]. Nadir platelet values often are <20,000/mL^3^ and the clinical manifestations range from petechia to more severe presentations, such as microangiopathic hemolytic anemias. Specific treatment is rarely needed in DITP, as it typically resolves within a few days of drug discontinuation [16]. Thymoquinone is a compound that is related to quinine and it is possible that they share a similar mechanism that leads to thrombocytopenia, although less literature is available on thymoquinone [10,11]. 

Evening primrose oil, an oil rich in γ-linolenic acid and omega-6-essential fatty acids, has been studied for its ability to affect platelet function. Studies in humans, rabbits, and rats have shown a decrease in platelet aggregation after ingestion of evening primrose oil [17-19]. Evening primrose oil’s anti-platelet effect may stem from its ability to inhibit thromboxane B2, increase synthesis of other prostaglandins with weaker aggregation effects, and reduce hyperlipidemia [17-19]. Although there is less literature on evening primrose oil's effects on platelet count, one study in rabbits reported a significant decrease in platelet count after ingestion of evening primrose oil capsules for 60 days [3]. It has also been reported that evening primrose oil may increase the risk of bleeding in those concurrently taking anti-thrombotics [20]. 

## Conclusions

In this case report, we highlight the role that black seed and primrose oil might have had in the patient’s clinical presentation. The patient’s thrombocytopenia was likely multifactorial with postoperative thrombocytopenia and consumptive thrombocytopenia as contributors. However, this acute drop in platelets was more significant compared to the typical values for postoperative and DVT/PE associated consumptive thrombocytopenia. The work-up for other causes of thrombocytopenia was largely negative, namely HIT, viral, thyroid, and autoimmune etiologies. Given black seed and evening primrose oil’s effects on platelets, we propose that the use of herbal supplements also contributed to the development of severe thrombocytopenia in the postoperative setting. Therefore, it is important to consider the contribution of herbal supplements when evaluating and treating a patient with postoperative and consumptive thrombocytopenia, especially when the work-up for other secondary causes is negative. 
